# Supplementary material for: Low CO2 levels of the entire Pleistocene epoch
Source: Nat Commun. 2019 Sep 25;10:4342. doi: 10.1038/s41467-019-12357-5 (PMC6761161; doi:10.1038/s41467-019-12357-5)
Supplement: Supplementary file 1 — Supplementary Information [file 41467_2019_12357_MOESM1_ESM.pdf]

Supplementary information for Low CO_2 levels of the entire Pleistocene Epoch by Da et al.

Supplementary Figure 1. Map shows the location of the Chinese Loess Plateau (yellow area), and the studied site of the Luochuan section (red star).

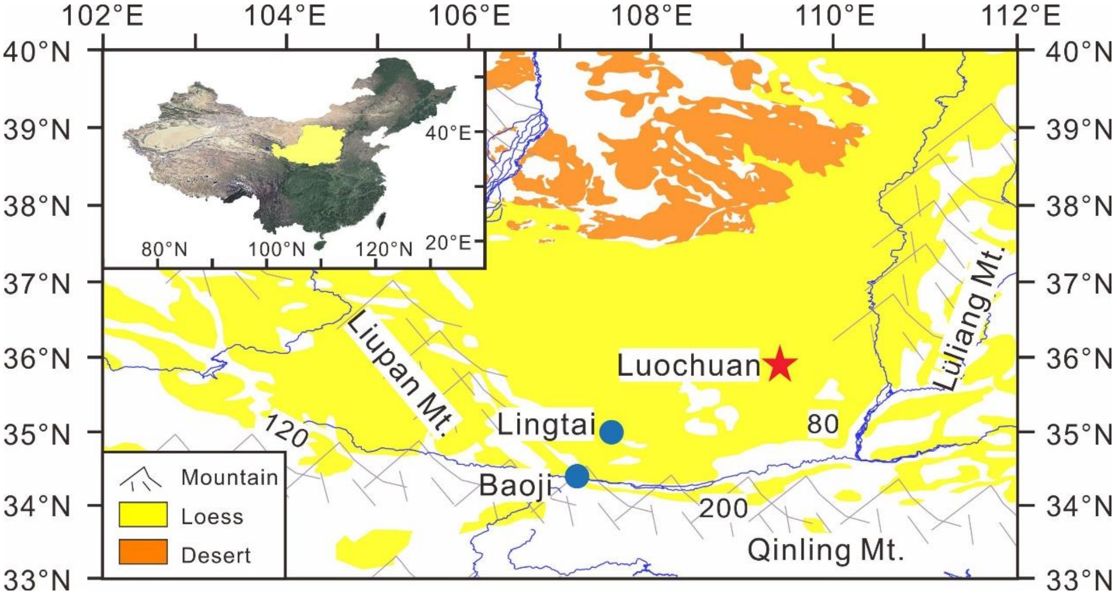

Supplementary Figure 2. Magnetic susceptibility, calcite and dolomite content of loess-paleosol sequences from Luochuan section. Each paleosol unit corresponds to specific marine isotope stage(s), as indicated by the stacked benthic $\delta^{18}O$ data[1].

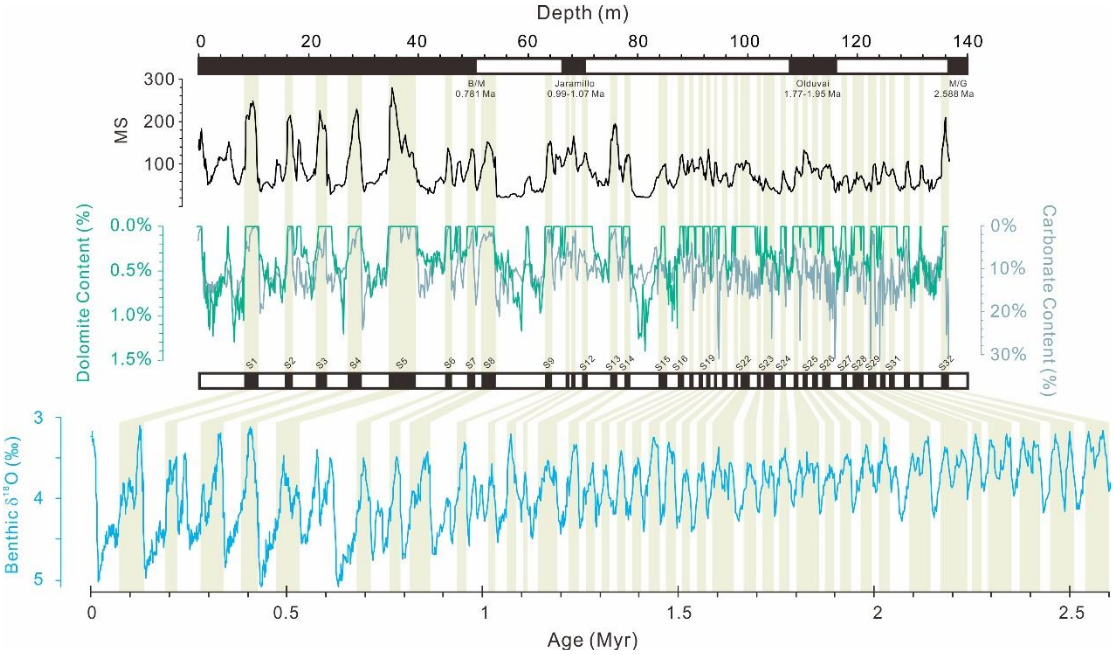

Supplementary Figure 3. Comparison of bulk paleosol carbonate and calcite nodule $\delta^{13}C$. **a** $\delta^{13}C$ values of bulk paleosol carbonates from Luochuan section in this study. **b** $\delta^{13}C$ of carbonate nodules from Lingtai and Baoji sections[2]. Error bars represent standard deviations of measured $\delta^{13}C$ values within the same paleosol units.

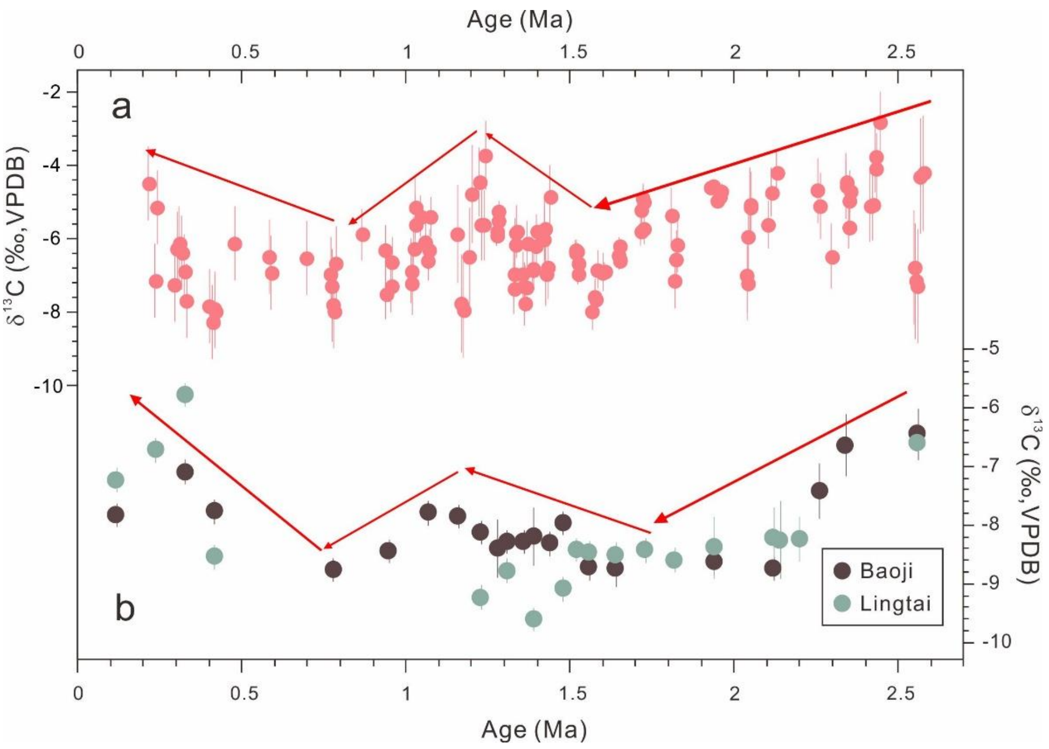

Supplementary Figure 4. Histograms of the mean relative differences (χ) between calculated and ice-core CO_2 in the test subset. The numbers of samples in the training subset (n) were set to 11, 12, 15 and 18 respectively. Red lines are probability density functions.

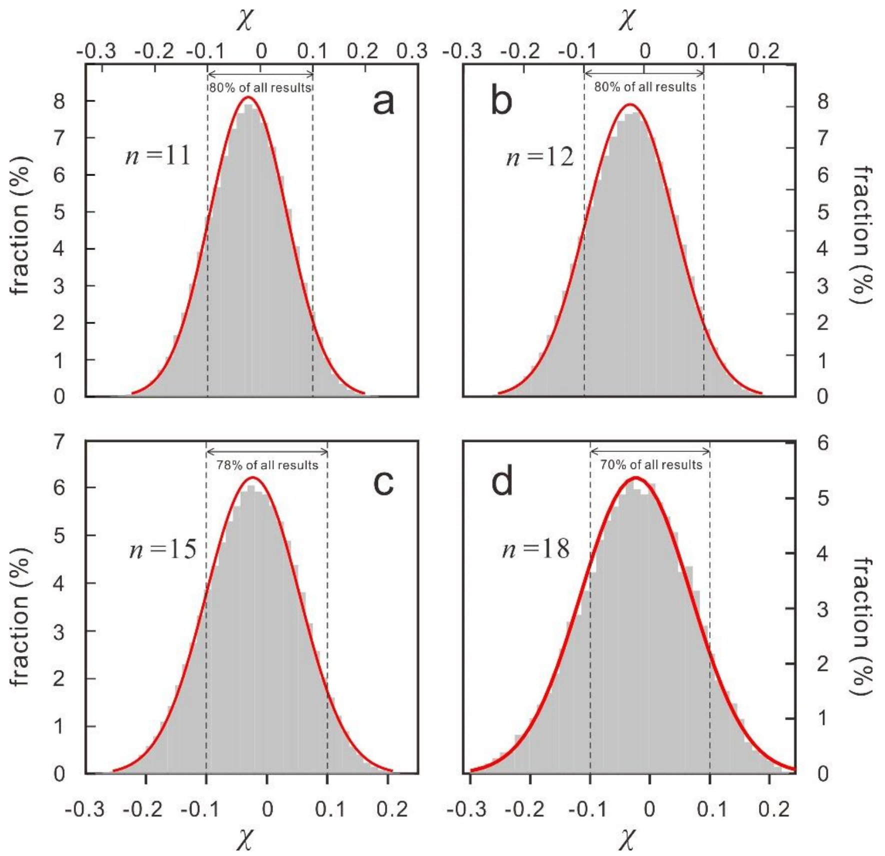

Supplementary Note 1

To assess the MS-S(z) relationship, S(z) values were first calculated through the reversed paleobarometer equation, using measured $\delta^{13}C_c$ and $\delta^{13}C_{SOM}$ of paleosol samples of the last 800 ky, as well as ice core pCO_2 data. Mean pCO_2 levels of each interglacial episode were used to back-calculate S(z), and errors associated with pCO_2 were assigned based on the standard deviations of pCO_2 values during each interglacial episode. Similar to the PBUQ program, errors associated with S(z) were propagated based on Monte Carlo simulations, which account for errors of all input variables (i.e. $\delta^{13}C_c$, $\delta^{13}C_{SOM}$, $\delta^{13}C_a$, T and pCO_2). The results yield median S(z) levels, as well as 16th and 84th percentiles of S(z) values ($\pm 1\sigma$).

The MS-S(z) regression equation was then generated, and errors associated with input S(z) values were taken into consideration. Specifically, we used the Gaussian error propagation, which is generalized as:

$$\delta f_{(x1,x2,\ldots xi)} = \sqrt{\Sigma(\frac{\partial f}{\partial x_i})^2 \partial x_i^2} \qquad [1]$$

The MS-S(z) regression equation used in this study can be expressed as:

$$S(z) = a \times MS + b \qquad [2]$$

where a and b represent the slope and intercept of the linear regression line (a = 2.66±0.44, b = 114.9± 71.1). Since MS was directly measured with high accuracy (<5%), analytical errors associated MS were ignored during subsequent error propagation. Applying Eq. 1 and we have:

$$\delta_{S(z)} = \sqrt{\left(\frac{\partial S(z)}{\partial a}\right)^2 \times (\delta_a)^2 + \left(\frac{\partial S(z)}{\partial b}\right)^2 \times (\delta_b)^2} = \sqrt{(0.44 \times MS)^2 + 71.1^2} \qquad [3]$$

Note that this error synthesizes the errors on the slope, the intercept of the regression, and varies as a function of the independent variable MS, thus different from the standard error of the regression. The standard errors ($\pm 1\sigma$) of S(z) were then applied to the PBUQ program for further error propagation of pCO_2.

Supplementary Note 2

To further verify potential variations of pCO_2 across the MPT, we converted our median paleosol-based pCO_2 distributions into median CO_2 change. Specifically, we use post-MPT (i.e. 0.8–0.4 Ma) as a reference time period for normalization as CO_2 data are known from ice cores; our median pCO_2 values

as well as 1σ during the MPT (n = 16) and pre-MPT (n = 21) were then divided by the mean CO_2 value of post-MPT interglacials to derive median factor CO_2 change ratios. Smoothed curve of factor CO_2 change (red curve in Fig. 4a) was generated using the locally weighted polynomial regression (LOESS) available from PAST freeware (http://folk.uio.no/ohammer/past/), which places higher significance on clustered data points more than discrete ones. A 0.3 smoothing factor is applied to capture the long-term trend of CO_2.

Moreover, we applied Monte Carlo simulations to calculate factor CO_2 change among the three time periods (i.e. MPT/pre-MPT, MPT/post-MPT, pre-MPT/post-MPT). First of all, values of $[CO_2]_{atm}$ were randomly chosen from normal distributions defined by means and standard deviations of all $[CO_2]_{atm}$ data from the three time periods. The three ratios were calculated during each of 10,000 iterations, the resulting distributions of which were then used to plot probability density functions (PDFs) and cumulative distribution functions (CDFs).

Supplementary Note 3

To evaluate the relative importance of each input from the paleosol barometer on $[CO_2]_{atm}$, we rearranged Equation 1. $\delta^{13}C_s$ can be solved by $\delta^{13}C_c$ through a temperature-dependent fractionation factor[3] which is expressed as

$$\delta^{13}C_s = \frac{\delta^{13}C_c+1000}{\frac{(11.98-0.12*T)}{1000}+1} - 1000 \qquad [3]$$

where T represents carbonate crystallization temperature. An approximate but much simpler equation is

$$\delta^{13}C_s = \delta^{13}C_c - (11.98 - 0.12\times T) \qquad [4]$$

substitute $\delta^{13}C_s$ in Equation 1 with $\delta^{13}C_c$ through equation 3, we obtain

$$pCO_2 = S(z)\times\frac{(\delta^{13}C_c-1.0044\delta^{13}C_r-16.38+0.12\times T)}{(\delta^{13}C_a-\delta^{13}C_c+11.98-0.12\times T)} \qquad [5]$$

Equation 4 shows that pCO_2 is controlled by five parameters: soil-respired CO_2 concentration at depth z during the time of carbonate precipitation (S(z)), carbon isotopic composition of atmospheric CO_2 ($\delta^{13}C_a$), pedogenic carbonate ($\delta^{13}C_c$) and soil-respired CO_2 ($\delta^{13}C_r$), as well as the formation temperature of pedogenic carbonate (T). $\delta^{13}C_a$ is eliminated during subsequence derivations as it remains as a constant (-6.5‰) through the study interval (11).

To examine the sensitivity of pCO_2 to the other three factors, one way is by computing

derivatives such as $S_i=\partial Y/\partial X_i$, where Y is an output and X_i is an input factor. However, the relationship between pCO_2 and each factor is not monotonic. For instance,

$$S_{\delta^{13}C_c} = \frac{\partial pCO_2}{\partial \delta^{13}C_c} = \frac{S_{(z)}\times(\delta^{13}C_c+0.12\times T-1.0044\times\delta^{13}C_{SOM}-16.38)}{(\delta^{13}C_c-5.48+0.12\times T)^2} - \frac{S_{(z)}}{(\delta^{13}C_c-5.48+0.12\times T)} \quad [6]$$

Not only $\delta^{13}C_c$ appears in the derivative, other factors are also introduced. This again shows the interdependence among the different factors. In order to examine the net effects of each factor on S(z) considering the interdependence, we utilized the SIMLAB software (https://ec.europa.eu/jrc/en/samo/simlab). Specifically, input parameters were constrained by the value and associated ±1σ error determined from the mean values and standard deviations of corresponding parameters in the dataset of this study. For instance, the mean $\delta^{13}C_c$ value of bulk soil carbonate is -6.2‰, and the mean standard error of $\delta^{13}C_c$ is 0.6‰ based on all the measurements in this study. Therefore, -6.2 ± 0.6‰ defines the normal distributions of the input $\delta^{13}C_c$. Using the clumped isotope thermometer, previous research based on Miocene-Pliocene calcite nodules across the CLP region have determined the formation temperature of pedogenic carbonate is generally 1-2 °C lower than modern day summer air temperature[4]. In this respect, we assigned a -1.5 °C correction of modern-day summer air temperature (JJAS) in Luochuan (i.e. 18.4°C), to represent the formation temperature in this study. An error range of ±3 °C is applied for the generation of the normal distribution. Then a Monte Carlo simulation was made based on the normal distributions of the inputs and 10,000 results were generated to calculate the sensitivity of each factor. The distributions of all factors were truncated at percentiles [10, 90] to ensure positive S_i values.

	mean	1σ	First order effect	Total order effect
S(z) (ppm)	407	74	0.2582	0.2984
$\delta^{13}C_c$ (‰)	-6.2	0.6	0.3372	0.3870
$\delta^{13}C_{SOM}$ (‰)	-24.4	0.7	0.2073	0.2299
T (°C)	18.4	3	0.1245	0.1553

Table. S1 The mean value and 1σ error of each input, as well as the first order and total order effect of each input parameter.

As shown in Table S1, pCO_2 is mostly sensitive to variations of $\delta^{13}C_c$, and relatively insensitive to temperature. The result is consistent with previous work[5], indicating that the clumped isotope thermometer is unlikely to improve the uncertainty of pCO_2 calculated from the paleosol barometer.

Supplementary References

1. Lisiecki, L. E. & Raymo, M. E. A Pliocene-Pleistocene stack of 57 globally distributed benthic $\delta^{18}O$ records. *Paleoceanography* 20 (2005).
2. Da, J., Zhang, Y. G., Wang, H., Balsam, W. & Ji, J. An Early Pleistocene atmospheric CO_2 record based on pedogenic carbonate from the Chinese loess deposits. *Earth Planet. Sci. Lett.* 426, 69-75 (2015).
3. Romanek, C. S., Grossman, E. L. & Morse, J. W. Carbon isotopic fractionation in synthetic aragonite and calcite: Effects of temperature and precipitation rate. *Geochim. Cosmochim. Acta* 56, 419-430 (1992).
4. Suarez, M. B., Passey, B. H. & Kaakinen, A. Paleosol carbonate multiple isotopologue signature of active East Asian summer monsoons during the late Miocene and Pliocene. *Geology* 39, 1151-1154 (2011).
5. Breecker, D. Improving paleosol carbonate-based estimates of ancient atmospheric CO_2. *Geochem. News* 144 (2010).
